# The Activation Status of the TGF-β Transducer Smad2 Is Associated with a Reduced Survival in Gastrointestinal Cancers: A Systematic Review and Meta-Analysis

**DOI:** 10.3390/ijms20153831

**Published:** 2019-08-05

**Authors:** Ilaria Girolami, Nicola Veronese, Lee Smith, Maria G. Caruso, Rosa Reddavide, Gioacchino Leandro, Jacopo Demurtas, Alessia Nottegar

**Affiliations:** 1 Department of Diagnostics and Public Health, Section of Pathology, University of Verona, 37134 Verona, Italy; 2 National Institute of Gastroenterology “S. de Bellis”, Research Hospital, Castellana Grotte, 70013 Bari, Italy; 3 The Cambridge Centre for Sport and Exercise Sciences, Anglia Ruskin University, Cambridge CB1 1PT, UK; 4 Primary Care Department, Azienda USL Toscana Sud Est, 58100 Grosseto, Italy; 5 Department of Diagnostics, Section of Pathology, San Bortolo Hospital, 36100 Vicenza, Italy

**Keywords:** Smad2, TGF-β, pSmad2, phosphorylation, signaling

## Abstract

Aberrant function of Smad2, a crucial member of transforming growth factor beta (TGF-β) signaling, is associated with the development of malignancies, particularly in the gastrointestinal district. However, little is known about its possible prognostic role in such tumor types. With the first meta-analysis on this topic, we demonstrated that the lack of the activated form of Smad2 (phosphor-Smad2 or pSmad2), which was meant to be the C-terminally phosphorylated form, showed a statistically significant association with an increased risk of all-cause mortality in patients with gastrointestinal cancers (RR, 1.58; 95% CI, 1.05–2.37, *p* = 0.029, I^2^ = 84%), also after having adjusted for potential confounders (RR, 1.65; 95% CI, 1.24–2.18; *p* < 0.001; I^2^ = 4%). This finding highlights the importance of the TGF-β signaling in this type of cancer. In this line, further studies are needed to explore more in depth this important molecular pathway, focusing also on potential therapeutic strategies based on its effectors or molecular targets.

## 1. Introduction

**Figure 1:**
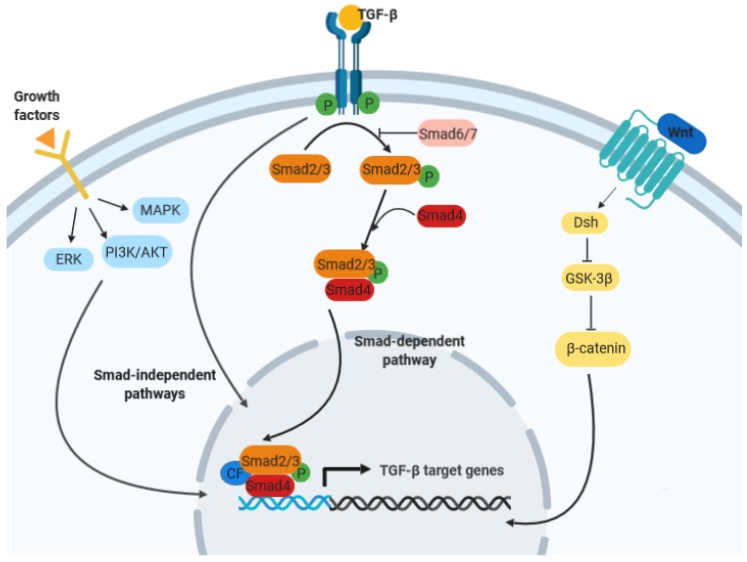
Schematic representation of the complex interactions involving TGF-β signaling and other important pathways in gastrointestinal tumors.

Transforming growth factor beta (TGF-β) signaling plays an essential role throughout development and later in adult homeostasis, interacting and coordinating different cell mechanisms. Notably, biological activities of TGF-β initiate with its binding to a heteromeric complex of two types of transmembrane receptors, namely type I and type II receptors; TGF-β occupancy induces an association between the type I and II receptors, causing the phosphorylation of the type I receptor by the constitutively active type II receptor [1,2,3,4,5,6]. The phosphorylated type I receptor then triggers activation of important cofactors, namely Smad2 and Smad3, by phosphorylating their C-terminal serine residues. Activated Smad2 and Smad3 form heteromeric complexes with Smad4 and other mediators, and translocate into the nucleus, forming a transcription complex with other cofactors, as well as participating also in the expression of target genes [1,2,3,4,5,6] (Figure 1).

Aberrant function of members of the TGF-β superfamily is associated with a wide range of human diseases, including cancer. Particularly, alterations in the activation of the protein Smad2, which is the product of the homonymous gene located on the q21.1 of the chromosome 18 (genomic location: chr18:47,808,957-47,931,193; www.genecards.org, last access 07/22/2019), appear very important in tumorigenesis [7,8,9,10,11,12]. 

The most important effect of TGF-β mediated by pSmad2 in cancer regards the loss of the role of tumor-suppressor, with a change to a pro-tumorigenic action for tumor progression. The pro-cancer progression consequences of aberrant TGF-β pathway signaling involves stromal remodeling, promotion of tumor cell plasticity (facilitating migration, invasion and metastasis), neo-angiogenesis and immunosuppression with loss of immunosurveillance [7,8,9,10,11,12]. pSmad2 expression is commonly used to describe the degree of TGF-β signaling activation, and nuclear pSmad2 is considered a good marker of activated TGF-beta signaling [13]. The expression of nuclear pSmad2 has been mainly investigated with immunohistochemistry (IHC), using different cut-off levels in varying studies to establish absent, low or high expression [13,14,15,16]. 

However, to further complicate the TGF-β landscape, recent evidence has indicated that the activation of this signaling pathway via Smad2, above all in late stages tumors, may promote tumorigenesis activating the epithelial-to-mesenchymal transition [17]. This dual role of TGF-β/Smad2 pathway renders its comprehension as a possible prognostic moderator in cancer more difficult. Moreover, the literature lacks a comprehensive summary on this topic. Given that this pathway is very important, among others, in gastrointestinal cancers [10,11,12], we decided to study the possible prognostic role of Smad2 in this type of tumor with the first systematic review and meta-analysis on this topic.

## 2. Results

### 2.1. Search Results

Altogether, 1197 non-duplicated articles were identified through the literature search. After excluding 1171 articles based on title/abstract review, 26 articles were retrieved for full text review and, following the application of the inclusion criteria, six unique articles were considered as eligible for the meta-analysis (Figure S1) [18,19,20,21,22,23].

### 2.2. Study and Patient Characteristics

The studies included in this meta-analysis regarded patient-cohorts from Asia (four studies) [18,19,20,22] or Europe (two studies) [21,23] and were equally divided among the most important gastrointestinal cancers, investigating indeed esophageal tumors (two studies) [18,19], gastric cancer (two studies) [20,22], and colorectal cancer (two studies) [21,23] (Table S1). 

Altogether, the studies followed-up 890 patients, 393 (44.2%) of which were pSmad2-. The mean Newcastle-Ottawa Scale (NOS) score was 8.5 points (range: 8–9), with no manuscript considered as at high risk of bias (Table S2). All studies reported adjusted analyses, but for one the specific values of pSmad2 after multivariable analysis were not available [18]; the median number of confounders considered in the multivariate analysis in the remaining studies was 6.4 (Tables S1 and S3). There were no statistically significant differences between pSmad2− and pSmad2+ groups of patients regarding gender, TNM stage or tumor grading. All data presented in this meta-analysis were derived from studies that have taken into account only the C-terminally phosphorylated Smad2. 

### 2.3. Association between pSmad2- and pSmad2+ and Survival

Pooling data from five studies [18,19,20,21,22], there was an increased risk ratio (RR) of all-cause mortality in patients with pSmad2-, which was statistically significant (RR, 1.58; 95% CI, 1.05–2.37, *p* = 0.029, I^2^ = 84%) (Table 1).

**Figure 2:**
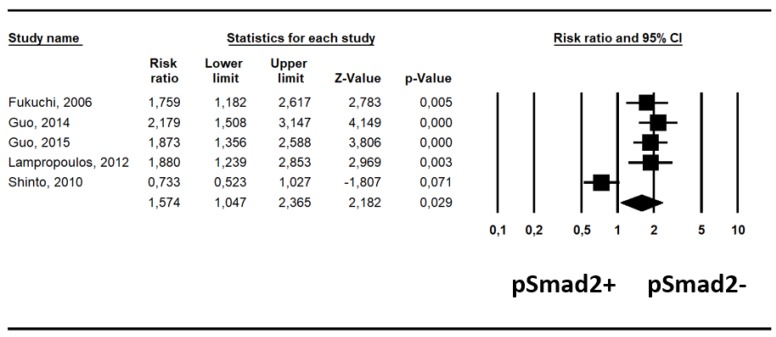
Forest-plot of relative risk of all-cause mortality: patients with pSmad2- have an increased risk of all-cause mortality, statistically significant (*p* = 0.029).

This association has also been highlighted by a forest-plot (Figure 2).

**Figure 3:**
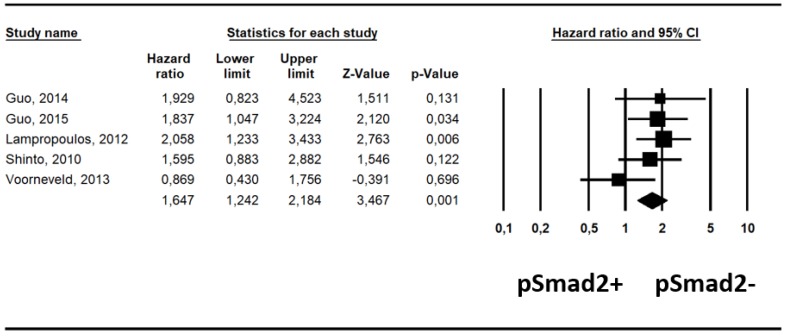
Forest-plot of adjusted risk of all-cause mortality: patients with pSmad2- have an increased risk of all-cause mortality, statistically significant after adjusting for potential confounders (*p* = 0.001).

In five studies reporting available adjusted data from multivariate analysis [19,20,21,22,23], after adjusting for a median of five covariates (range: 5–8) (Table S3), pSmad2- carried a significantly higher risk of all-cause mortality compared to pSmad2+, increasing its statistical significance (RR, 1.65; 95% CI, 1.24–2.18; *p* < 0.001; I^2^ = 4%) (Table 1, Figure 3).

### 2.4. Publication Bias and Meta-Regression Analyses

There was no risk of publication bias for both analyzed indexes, i.e., unadjusted RR for survival (Egger’s test, 8.62 ± 12.51, *p* = 0.54) and adjusted estimates (Egger’s test, −2.28 ± 2.83, *p* = 0.48). 

There was high heterogeneity only for (unadjusted) RR of all-cause mortality and, therefore we investigated if differences in TNM III-IV or G3 prevalence between Smad2+ and Smad2- could be significant moderators of the heterogeneity found. Neither the difference in stage, as measured by TNM III-IV between the two groups (slope = 0.005 ± 0.008; *p* = 0.55) or grading, measured as the prevalence in G3 cancers (slope = 0.02 ± 0.006; *p* = 0.09), were moderators of the heterogeneity. 

## 3. Discussion

The present meta-analysis investigating the prognostic role of pSmad2 in gastrointestinal cancers, found a statistically significant association between the absence of pSmad2+ (pSmad2-) and an increased risk of mortality. This finding appears robust also considering that the association of pSmad2- and risk of mortality has been confirmed using data from multivariate analyses. The independent prognostic value of pSmad2- was confirmed also observing that both cohorts of patients, pSmad2- vs. pSmad2+, showed no significant differences either in terms of TNM stage or of tumor grading. To further reinforce the reliability of the present finding, the risk of publication bias was not detected.

Smad2 is a specific intracellular mediator of TGF-β signaling and plays a pivotal role in the antiproliferative effects of this pathway [18]. A seminal paper in this field pointed out that the level of pSmad2 is inversely associated to histological differentiation in head and neck tumors [24], indicating a potential prognostic role of the loss of this marker as an indicator of poor prognosis. In our meta-analysis, pSMAD2- confirms its clinical value, being associated with a higher risk of mortality also considering potential confounders. However, the biological basis of this association is not well understood. 

First, it is important to consider that, in the TGF-β signaling, Smad2 is activated by phosphorylation upon activation of the TGF-β receptor [25]. Notably, in this meta-analysis we have considered studies that have taken into account only the C-terminally phosphorylated Smad2, since different sites of phosphorylation may result in totally different biological functions. The levels of pSmad2 may directly reflect TGF-β-induced growth inhibitory/anti-proliferative effects [18,26]. Unresponsiveness to TGF-β-induced growth inhibition as a direct result of the lack of pSmad2 may have different effects in cancer biology, from the stimulation of tumor development and growth to distant metastasization. Notably, the phosphorylation of Smad2 is the most important step for this factor to control its function, and this explains that the vast majority of cancers with an impaired Smad2 action have only a dysregulated Smad2 phosphorylation and do not present a matched *SMAD2* driver gene mutation [12,27]. Indeed, this gene has a low rate of mutations in gastrointestinal cancers, particularly if compared with its counterpart *SMAD4*, which is one of the most important driver genes in gastroentero-pancreatic tumors [28,29,30,31].

Another important aspect to be considered in interpreting the present results on the negative prognostic role of pSmad2- in gastrointestinal cancers regards the process of epithelial-to-mesenchymal transition (EMT). EMT is a cell mechanisms in which the epithelial elements lose their polarity and cell-to-cell contacts, undergo remodeling of the cytoskeleton with morphological modifications, acquiring also motility and migratory capacities [32]. Particularly, in cancer biology and in several cancer types, EMT is a fundamental process for tumor development and metastasization [33,34,35,36,37]. Particularly, EMT via TGF-β/Smad pathway has been investigated in different cancer types [38,39,40,41], where TGF-β emerges as a tumor suppressor in the early stages of tumorigenesis, whereas in later stages it may favor tumor progression and metastatization via EMT activation [17,21,42,43]. In later stages, indeed, tumor cells may undergo EMT through TGF-β signaling, becoming more invasive and with greater metastatic potential. TGF-β suppresses immune responses of non-transformed cancer cells, increasing neo-angiogenesis and leading to progression, metastasis and even to the unfavorable prognostic event of extranodal extension of nodal metastasis [17,21,42,43,44,45,46,47,48,49]. All these events may explain, at least partly, the biological reasons of the negative prognostic role of pSmad2- that has been demonstrated with this meta-analysis. The switching of TGF-β signaling from a tumor suppressor to a tumor promoter is essential to better understanding this specific step in the complex process of tumorigenesis. 

Although the finding of a correlation of the absence of pSmad2 expression with a worse prognosis seems to contradict the general assumption that enhanced TGF-beta signaling activity favors tumor progression in late tumor stages, one possible further explanation is that tumor progression is driven by pSmad3, rather than pSmad2, and that pSmad2 acts as an endogenous inhibitor of the tumor-promoting function of pSmad3.

In this line, it is also of importance considering the role of Smad3 in the complex interaction of TGF-β pathway with EMT. Smad3 indeed has been demonstrated as an important mediator in such interaction. For example, renal tubular epithelial cells deficient in Smad3 fail to undergo EMT in response to TGF-β or mechanical stress, and keratinocytes from Smad3–/– mice demonstrate reduced migration in response to TGF-β [50]. Compared with Smad3, Smad2 may play an antagonistic role in the EMT process in vivo: In fact, loss of Smad2 has been frequently shown in human skin cancers, and Smad2 deficiency in keratinocytes promotes EMT accelerating skin tumorigenesis [50]. This has been explained by increased binding of the Smad3/4 complex to the promoter of the *SNAIL* gene and by increased SNAIL expression in the absence of Smad2, thus enhancing the progression of EMT [50]. In a similar way, also highlighting that Smad2 is important in different tissues, Smad2–/– hepatocytes appear mesenchymal and migrate faster than wild-type cells, whereas Smad3–/– hepatocytes retain their epithelial features [50].

Although the results of this systematic review with meta-analysis appears reliable, we recognize in it also some limitations, which are largely reflected by those within the primary studies. First, the design of the studies included in the present review were retrospective; moreover, in these studies, data about other co-morbidities (like cardio-vascular diseases) were not specifically considered, but it is known that such comorbidities also play an important clinical role in patients with cancer. A final limitation includes the high heterogeneity found for the unadjusted relative risk for all-cause mortality. However, using data from multivariable models, the heterogeneity became low and at the same time the statistical significance of the present results was preserved, further corroborating the reliability of the finding.

In conclusion, with this meta-analysis it has been demonstrated that lack of pSmad2 is strongly associated with an increased risk of mortality in gastrointestinal cancers. This finding highlights the importance of the TGF-β signaling in cancer. Further studies are now needed to explore more in depth this molecular pathway and also possible therapeutic strategies, taking into account its potential effectors or molecular targets.

## 4. Materials and Methods 

This systematic review adhered to the Meta-analyses Of Observational Studies in Epidemiology (MOOSE) guidelines and Preferred Reporting Items for Systematic Reviews and Meta-Analyses (PRISMA) statement, following a predetermined protocol [51,52]. 

### 4.1. Inclusion and Exclusion Criteria

Studies were eligible for inclusion upon meeting the following criteria: (1) a prospective cohort or retrospective study design; (2) it contained a comparison of prognostic factors between positive vs. negative pSmad2 with immunohistochemistry; (3) it considered only C-terminally phosphorylated Smad2; (4) a diagnosis of conventional gastrointestinal cancers; (5) it contained data about mortality/recurrence of disease; (6) it was published in a peer review journal or published abstract. Articles in any language were considered. 

Exclusion criteria were: (1) no presence of specific data on pSma2, (2) no clear standards of immunohistochemical protocol and its interpretation, (3) no data about prognostic parameters in the title/abstract, (4) no comparison between positive vs. negative pSmad2 patients, and (5) in vitro or animal studies.

We selected research papers that investigated Smad2 with immunohistochemistry since this is the best method to study the effective Smad2 function, given that *SMAD2* mutations are very rare in gastrointestinal cancers and do not represent a reliable indicator of Smad2 activity.

### 4.2. Data Sources and Literature Search Strategy

Two investigators (I.G., N.V.) independently searched PubMed, Embase and SCOPUS until 05/31/2019. The search terms used in PubMed included combinations of the following keywords: (SMAD2 OR JV18 OR JV18-1 OR MADH2 OR MADR2 OR hMAD-2 OR hSMAD2) AND (cancer OR neoplasm OR neoplasia OR carcinoma OR adenocarcinoma) AND (mortality OR mortalities OR fatality OR fatalities OR death* OR survival OR “hazard ratio” OR HR OR “relative risk” OR RR OR progression OR recurrence). A similar research was made in SCOPUS and Embase. We considered the reference lists of all included articles and of previous related reviews. 

### 4.3. Study Selection

Following the searches as outlined above, after removal of duplicates, two independent reviewers (I.G., L.S.) screened titles and abstracts of all potentially eligible articles. The two authors applied the eligibility criteria, considered the full texts, and a final list of included articles was reached through consensus with a third author (A.N.).

### 4.4. Data Extraction 

Two authors were involved in data extraction in a standardized Microsoft Excel database. Specifically, one author (N.V.) extracted data from the included articles and a second independent author (A.N.) validated the data. For each article, the following information was extracted: Authors, year of publication, country, exclusion criteria, other genes/proteins abnormalities that have been studied in the specific work, number of participants and gender, age, pathologic TNM stage, tumor grading, type of IHC analysis (whole section vs. tissue micro-array), number of adjustments in survival analyses and duration of follow-up. 

### 4.5. Outcomes

The primary outcome was the number of deaths after treatment during the follow-up period in patients with positive vs. negative pSmad2. The secondary outcome was the risk, adjusted for the maximum number of confounders available in each paper, regarding the same outcome, taking those with positive pSmad2 as reference.

### 4.6. Assessment of Study Quality

We used the Newcastle-Ottawa Scale (NOS) to evaluate study quality [53]. The NOS provides an assessment of the methodological quality of observational studies and its content validity and reliability have been widely assessed [54]. Included studies are assessed on 8 items across three key areas: selection of the participants, comparability of the participants and outcomes. Two authors (A.N., N.V.) completed the NOS and each study received an overall score for methodological quality of up to 9 points with a score of ≤5 (out of 9) indicating high risk of bias. 

### 4.7. Data Synthesis and Statistical Analysis 

All analyses were performed using Comprehensive Meta-Analysis (CMA) 2 (Biostat, Englewood, NJ, USA). In our primary analyses, pooled risk ratios (RRs) and 95% confidence intervals (CIs) of risk of mortality between positive pSmad2 vs. negative pSmad2 were calculated using DerSimonian-Laird random-effects models [55]. In secondary analyses, pooled hazard ratios (HRs) with 95% CIs adjusted for the maximum number of covariates available in the articles were also calculated for providing additional information if the relationship between pSmad2 status and survival was influenced by potential confounders. Heterogeneity across studies was assessed by the I^2^ metric and chi square statistics [56]. In the presence of significant heterogeneity (*p* < 0.05) after removing outlier studies, a series of meta-regression analyses according to pSmad2 status and each of prognostic parameters were considered. Finally, publication bias was investigated for the primary meta-analysis with a visual inspection of funnel plots coupled with the Egger bias test [57]. 

## Figures and Tables

**Table 1:** Risk ratios (unadjusted and adjusted) for all-cause mortality (ACM) of patients with gastrointestinal cancers, based on pSmad2- vs. pSmad2+ status.

Parameter	N Studies	Risk Ratio (95% CI)	*p* Value	Heterogeneity (I2%); tau2	Egger Test ± SE (*p* Value)
Unadjusted	5	1.58 (1.05–2.37)	0.029	84%, *p* < 0.0001	8.62 ± 12.51 (*p* = 0.54)
Adjusted	5	1.65 (1.24–2.18)	<0.001	4%; *p* = 0.38	−2.28 ± 2.83 (*p* = 0.48)

Abbreviations: ACM: All-cause mortality; CI: Confidence intervals; SE: Standard error.

## Supplementary Materials

Supplementary materials can be found at https://www.mdpi.com/1422-0067/20/15/3831/s1. Figure S1. PRISMA checklist for this meta-analysis; Table S1. Characteristics of The Studies According to the Expression of SMAD2; Table S2. Methodological Quality of Cohort Studies Included in the Meta-Analysis; Table S3. Type and number of adjustments (in addiction of pSMAD2 status) for each study.

## Abbreviations

TGF-β	Transforming growth factor beta
IHC	immunohistochemistry
TNM	Tumor Nodes Metastasis staging system
G	Grading
MOOSE	Meta-analyses Of Observational Studies in Epidemiology guidelines
PRISMA	Preferred Reporting Items for Systematic Reviews and Meta-Analyses
