# Supplementary material for: The Activation Status of the TGF-β Transducer Smad2 Is Associated with a Reduced Survival in Gastrointestinal Cancers: A Systematic Review and Meta-Analysis
Source: Int J Mol Sci. 2019 Aug 5;20(15):3831. doi: 10.3390/ijms20153831 (PMC6695973; doi:10.3390/ijms20153831)
Supplement: Supplementary file 1 [file ijms-20-03831-s001.pdf]

**Figure S1. PRISMA checklist for this meta-analysis.**

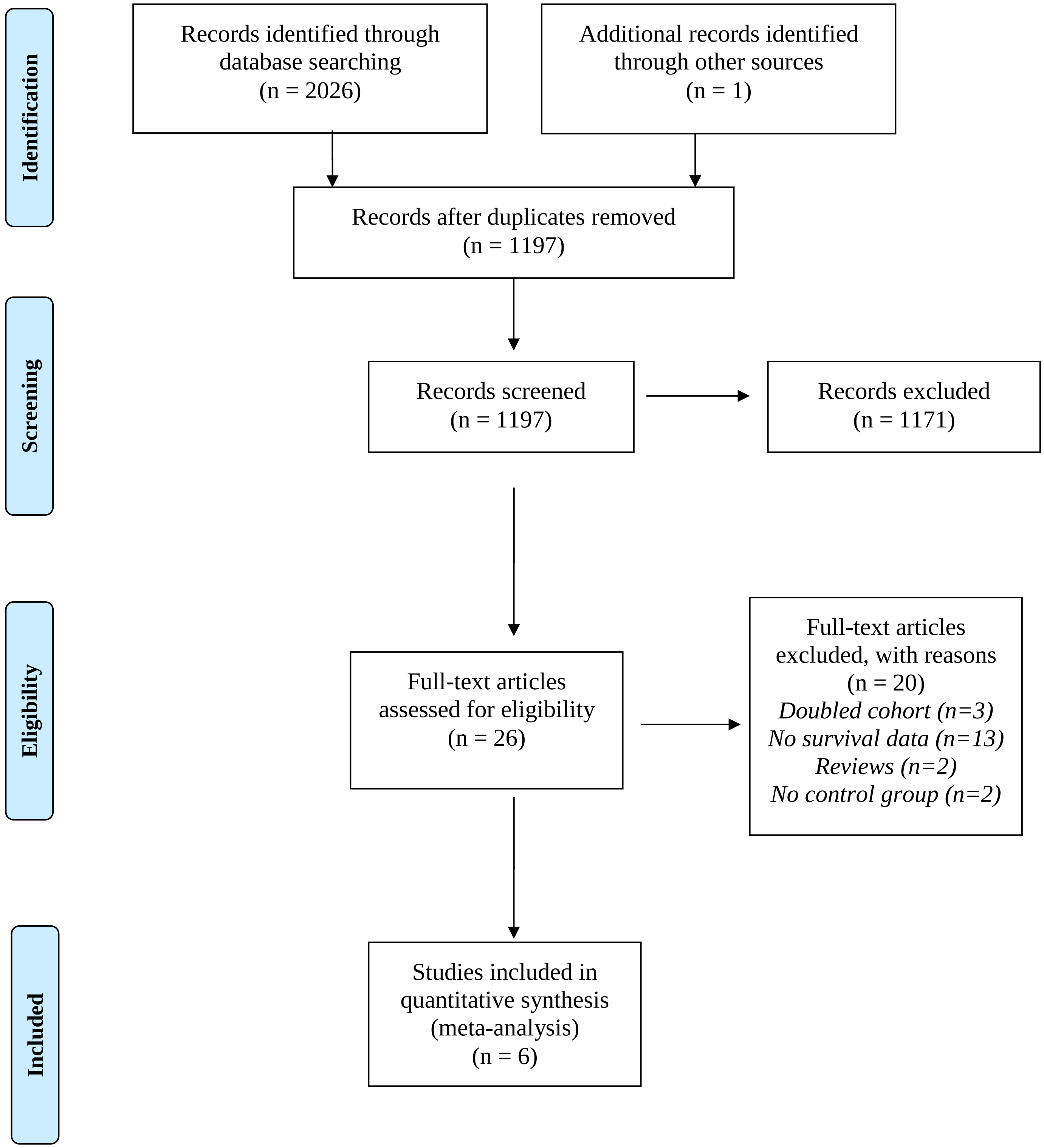

**Table S1.** Characteristics Of The Studies According To The Expression Of SMAD2.

| | | | | pSMAD2- | | | | | pSMAD2+ | | | | | | | |
|---|---|---|---|---|---|---|---|---|---|---|---|---|---|---|---|---|
| **Study Author, Year (Country)** | **Type of cancer** | **Exclusion criteria** | **Other genes/ proteins abnormalities** | **Number of participants** | **N. of females (%)** | **Mean Age ± SD** | **TNM stage at baseline (number, %)** | **Tumor Grading** | **Number of participants** | **N. of females (%)** | **Mean Age ± SD** | **TNM stage at baseline (number, %)** | **Tumor Grading** | **Methods of SMAD2 analysis** | **Number of adjustments** | **Mean Follow-up period (months)** |
| **Fukuchi, 2006 (Japan)** | Esophageal SCC | NT | Smad2, Smad3 | 29 | 13,8% | 60.2 ± 1.6 | I-II: 38% III-IV: 62% | G1-2: 62% G3: 38% | 51 | 13,7% | 62.4 ± 1.1 | I-II: 66.6% III-IV: 33.3% | G1-2: 80.4% G3: 19.6% | Whole section IHC[1] | 5 | >60 |
| **Guo, 2014 (China)** | Esophageal SCC | NS | FBXO32, Smad4 | 77 | 28.6% | NS | I-II: 45.5% III-IV: 54.5% | G1-2: 53.2% G3: 46.8% | 55 | 30.9% | NS | I-II: 67.3% III-IV: 32.7% | G1-2: 70.9% G3: 29.1% | Whole section IHC[2] | 8 | Range 18-78, median 66 |
| **Guo, 2015 (China)** | GCA | Lesion not centred in gastroesophageal junction | FBXO32, Smad4 | 80 | 22.5% | NS | I-II: 36.3% III-IV: 63.7% | G1-2: 71.3% G3: 28.7% | 59 | 20.3% | NS | I-II: 54.2% III-IV: 45.8% | G1-2: 86.4% G3: 13.6% | Whole section IHC[3] | 7 | Range 18-84, median 66 |
| **Lampropoulos, 2012 (Greece)** | CRC | NT | TGF-β, TGF-β R1, TGF-β R2, Smad4, E-cadherin | 56 | 44.6% | NS | I-II: 76.8% III-IV: 23.2% | NS | 139 | 48.2% | NS | I-II : 50.4% III-IV: 49.6% | NS | Whole section IHC[4] | 5 | Range 1-72, median 56.0±16.7 |

| | | | | | | | | | | | | | | | | |
|---|---|---|---|---|---|---|---|---|---|---|---|---|---|---|---|---|
| **Shinto, 2010 (Japan)** | Gastric cancer | NT | None | 72 | 25% | NS | I-II: 41.7% III-IV: 58.3% | G1-2: 45.8% G3: 54.2% | 63 | 38.1% | NS | I-II: 27% III-IV: 73% | G1-2: 23.8% G3: 76.2% | Whole section IHC[5] | 6 | >60 |
| **Voorneveld, 2013 (the Netherlands)** | CRC | Neoadjuvant radiotherapy | Smad4, pSmad1,5,8 | 79 | NS | NS | NS | NS | 130 | NS | NS | NS | NS | TMA-IHC[6] | 6 | 71 |
| **Total Studies (means, SDs and percentages are weighted with n values)** | **2 CRC, 2 gastric cancer, 2 esophageal cancer** | **-** | **-** | **393** | **27.7% (5 studies)** | **60.2 ± 1.6 (1 study)** | **TNM: (5 studies): I-II: 47.2%; III-IV: 52.8%; NA: 1 study** | **G1-G2: (4 studies) 57.7% G3: 42.3; NA: 2 studies** | **497** | **34.6% (5 studies)** | | **TNM: (5 studies): I-II: 51.8%; III-IV: 48.2%; NA: 1 study** | **G1-G2: (4 studies) 64% G3: 36%; NA: 2 studies** | **TMA-IHC: 1 study; Whole section IHC: 5 studies** | **range: 5-8** | **> 6.3 years** |

Notes: [1]phospho-Smad2 (Upstate Biotechnology, Lake Placid, NY, USA; dilution 1:50) and Smad2 (Transduction Laboratories, Lexington, KY, USA); [2]phospho-Smad2/3 (Santa Cruz Biotechnology Inc., Santa Cruz, Calif, dilution 1:200); [3]phospho-Smad2/3 (Ser423/425, Santa Cruz, San Diego, CA, USA, dilution 1:200); [4]phospho-Smad2/3 (Ser423/425, sc-11769, Santa Cruz Biotechnology, dilution 1:50); [5]phospho-Smad2 (Chemicon International, Temecula, CA, dilution 1:2000); [6]phospho-Smad2,3 (Cell Signaling Technology, Boston, MA, USA);

Abbreviations: CRC: colorectal cancer; GCA: gastric cardia adenocarcinoma; IHC: immunohistochemistry; NA: not available; NS: not specified; NT: neo-adjuvant treatments; SCC: squamous cell carcinoma; TMA-IHC: tissue micro-array immunohistochemistry; TNM: tumor, nodes, metastasis.

**Table S2.** Methodological Quality Of Cohort Studies Included In The Meta-Analysis*

| **First author, publication year** | **Representativeness of the exposed cohort** | **Selection of the unexposed cohort** | **Ascertainment of exposure†** | **Outcome of interest not present at start of study††** | **Control for important factor or additional factors†††** | **Assessment of outcome** | **Follow-up long enough for outcomes to occur††††** | **Adequacy of follow-up of cohorts** | **Total quality scores** |
|---|---|---|---|---|---|---|---|---|---|
| Fukuchi, 2006 | * | * | * | * | * | * | * | * | 8 |
| Guo, 2014 | * | * | * | * | ** | * | * | * | 9 |
| Guo, 2015 | * | * | * | * | ** | * | * | * | 9 |
| Lampropoulos, 2012 | * | * | * | * | ** | * | - | * | 8 |
| Shinto, 2010 | * | * | * | * | * | * | * | * | 8 |
| Voorneveld, 2013 | * | * | * | * | ** | * | * | * | 9 |

Original studies were analyzed in the quality assessment.

* A study could be awarded a maximum of one star for each item except for the item Control for important factor or additional factor. The definition/explanation of each column of the Newcastle-Ottawa Scale is available at http://www.ohri.ca/programs/clinical_epidemiology/oxford.htm.

† For this index, one star was given if in Method section the SMAD2 expression was assessed with immunohistochemistry (IHC), or with whole section-IHC, or, in case of the use of tissue microarray-IHC, using at least 2 cores per case.

†† Being outcome of interest mortality, we took as outcome of interest for assessment of quality if the overall survival or the recurrence rate was assessed.

††† A maximum of 2 stars could be awarded for this item. Studies that controlled their survival analyses for at least two confounders received one star, whereas studies that assessed and described the expression of also SMAD3, an additional star.

†††† A cohort study with a mean/median follow-up time ≥5 y (60 months) takes one star.

**Table S3.**Type and number of adjustments (in addiction of pSMAD2 status) for each study

| First author, publication year | Adjustments | Maximum number of adjustments |
|---|---|---|
| Fukuchi, 2006 | pT, G, pN, M, TNM-S | 5 |
| Guo, 2014 | TNM-S, pN, M or recurrence, Smad4 expression, depth of invasion, family history of upper GI cancer, FBXO32 expression, FBXO32 methylation | 8 |
| Guo, 2015 | TNM-S, family history of upper GI cancer, Smad4 expression, FBXO32 expression, FBXO32 methylation, age, gender | 7 |
| Lampropoulos, 2011 | TGF-β, TGF-β R1, TGF-β R2, Smad4, E-cadherin expression | 5 |
| Shinto, 2010 | Morphologic feature, differentiation (intestinal vs diffuse), pN (Japanese classification), peritoneal dissemination, lymphatic invasion, TNM-S | 6 |
| Voorneveld, 2013 | Age, Sex, G, Dukes stage, Smad4 expression, pSmad1,5,8 expression | 6 |

Abbreviations: CRT: chemo-radiotherapy; G: histologic grading; GI: gastrointestinal; M: distant metastasis; pN: lymph node status in pathologic TNM;  pT: tumor stage in pathologic TNM; R: radicalness of resection; TNM: tumor-node-metastasis staging system; TNM-S: TNM Stage.
